# Microbial Production of Retinyl Palmitate and Its Application as a Cosmeceutical

**DOI:** 10.3390/antiox9111130

**Published:** 2020-11-14

**Authors:** Bo Hyun Choi, Hee Jin Hwang, Ji Eun Lee, Soon Hwan Oh, Jae Sung Hwang, Bun Yeoul Lee, Pyung Cheon Lee

**Affiliations:** 1 Department of Molecular Science and Technology, Ajou University, World cup-ro, Yeongtong-gu, Suwon-si, Gyeonggi-do 16499, Korea; bohyunho0@ajou.ac.kr (B.H.C.); jinilucifer@ajou.ac.kr (H.J.H.); portejuio@ajou.ac.kr (S.H.O.); bunyeoul@ajou.ac.kr (B.Y.L.); 2 Department of Genetic Engineering & Graduate School of Biotechnology, College of Life Sciences, Kyung Hee University, Yongin-si, Gyeonggi-do 17104, Korea; jieun0405@khu.ac.kr (J.E.L.); jshwang@khu.ac.kr (J.S.H.)

**Keywords:** retinoid, retinyl palmitate, carotenoids, antioxidants

## Abstract

Chemically synthesized retinyl palmitate has been widely used in the cosmetic and biotechnology industry. In this study, we aimed to demonstrate the microbial production of retinyl palmitate and the benefits of microbial retinyl palmitate in skin physiology. A heterologous retinyl palmitate biosynthesis pathway was reconstructed in metabolically engineered *Escherichia coli* using synthetic expression modules from *Pantoea agglomerans*, *Salinibacter ruber*, and *Homo sapiens*. High production of retinyl palmitate (69.96 ± 2.64 mg/L) was obtained using a fed-batch fermentation process. Moreover, application of purified microbial retinyl palmitate to human foreskin HS68 fibroblasts led to increased cellular retinoic acid-binding protein 2 (CRABP2) mRNA level [1.7-fold (*p* = 0.001) at 100 μg/mL], acceleration of cell proliferation, and enhancement of procollagen synthesis [111% (*p* < 0.05) at 100 μg/mL], strongly indicating an anti-ageing-related effect of this substance. These results would pave the way for large-scale production of retinyl palmitate in microbial systems and represent the first evidence for the application of microbial retinyl palmitate as a cosmeceutical.

## 1. Introduction

Vitamin A derivatives include a number of related nutritional hydrophobic compounds, such as retinal, retinol, retinoic acid, and several retinyl esters. Vitamin A derivatives serve essential roles in aiding healthy vision, maintenance of the immune system, embryonic growth and development, and protection of epithelial tissues [1,2]. Vitamin A consists of three structural domains: a cyclic moiety, a polyene side chain, and a polar end group. β-carotene is converted in vivo into vitamin A derivatives (retinoids) through the addition of functional groups, such as a hydroxyl in retinol, an aldehyde in retinal, a carboxylic acid in retinoic acid, and an ester in retinyl ester [3].

**Figure 1:**
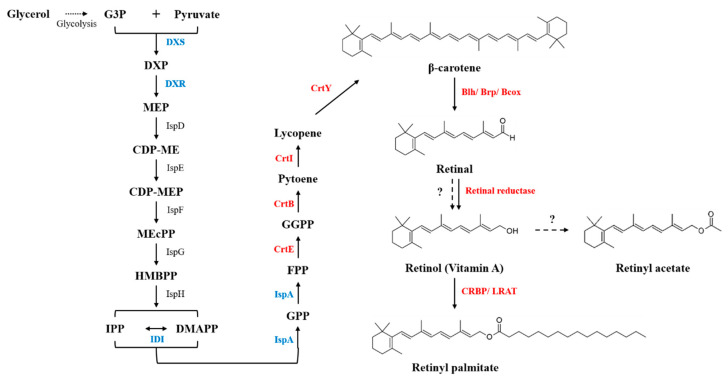
The reconstructed retinyl palmitate biosynthesis pathway in *Escherichia coli*. Endogenous enzymes subjected to overexpression are highlighted in blue. Heterogenous enzymes for the retinoid pathway are shown in red. Metabolite abbreviations: CDP-ME, 4-diphosphocytidyl-2-C-methyl-D-erythritol; CDP-MEP, 4-diphosphocytidyl-2-C-methyl-D-erythritol-2-phosphate; DMAPP, dimethylallyl diphosphate; DXP, 1-deoxy-D-xylulose-5-phosphate; FPP, farnesyl diphosphate; G3P, glycerol-3-phosphate; GGPP, geranylgeranyl diphosphate; GPP, geranyl diphosphate; HMBPP, 4-hydroxy-3-methyl-butenyl 1-diphosphate; IPP, isopentenyl diphosphate; MEcPP, 2-C-methyl-D-erythritol-2,4-cyclodiphosphate; and MEP, 2-C-methyl-D-erythritol-4-phosphate. The enzymes involved in this pathway include: Bcox, β-carotene 15,15′-oxygenase; Blh, bacteriorhodopsin-related protein-like homology protein; Brp, bacteriorhodopsin-related protein; CRBP, cytoplasmic retinol-binding protein; CrtB, phytoene synthase; CrtE, geranylgeranyl diphosphate synthase; CrtI, phytoene desaturase; DXR, 1-deoxy-D-xylulose-5-phosphate reductoisomerase; DXS, 1-deoxy-D-xylulose-5-phosphate synthase; IDI, isopentenyl diphosphate isomerase; IspA, farnesyl diphosphate synthase; IspD, 2-C-methyl-D-erythritol-4-phosphate cytidylyltransferase; IspE, 4-diphosphocytidyl-2-C-methyl-D-erythritol kinase; IspF, 2-C-methyl-D-erythritol-2,4-cyclodiphosphate synthase; IspG, 1-hydroxy-2-methyl-2-(E)-butenyl 4-diphosphate synthase; IspH, 4-hydroxy-3-methyl-2-(E)-butenyl 4-diphosphate reductase; LRAT, lecithin retinol acyltransferase. The dotted arrows represent unknown enzymatic reactions, while the question marks represent endogenous enzymes having functions in the corresponding reactions.

Retinoids are biosynthesized in several steps that are catalyzed by enzymes (Figure 1). The first step is the cleavage of β-carotene by β-carotene 15,15′-oxygenase (Bcox) for the synthesis of retinaldehyde, also known as retinal [4]. The next step is the oxidation or reduction of retinal for retinoic acid synthesis or retinol synthesis by retinal dehydrogenase or retinal reductase, respectively [5]. In the case of retinol biosynthesis, cytoplasmic retinol-binding protein (CRBP) accepts retinol for retinol storage and homeostasis in vivo before esterification for the synthesis of retinyl esters [6]. Finally, retinyl ester, a storage form of retinol, is synthesized via esterification of a fatty acyl group to the hydroxyl terminus of retinol; this reaction is catalyzed by lecithin retinol acyltransferase (LRAT) [7]. In the human body, the fatty acid moiety in retinyl esters can be palmitic acid, oleic acid, or linoleic acid [8].

Bcox has been characterized in many animal species, including chickens [9], humans [10], and mice [11]. After studies on animal Bcox, two types of microbial enzymes for converting β-carotene to retinal [12]—bacteriorhodopsin-related protein (Brp) and bacteriorhodopsin-related protein-like homology protein (Blh)—were identified in bacteria, including *Haloarcula marismortui* [13], *Halobacterium salinarum* [14], uncultured marine bacterium HF10 49E08 [15], and *Salinibacter ruber* [16]. In particular, the halophilic bacterium *S. ruber* has a retinal-based light-driven pump known as xanthorhodopsin, and retinal-related proteins of *S. ruber* have been proposed to catalyze the conversion of β-carotene to retinal [17]. Nonetheless, there is no biochemical evidence of these enzymatic activities in *S. ruber*.

Retinol exerts an anti-ageing activity on the skin; for example, retinoic acid induces epidermal thickening, inhibits induction of matrix metalloproteinases by UV light, and promotes collagen synthesis in photoaged skin [18], in addition to displaying antioxidant activity [7]. Due to its high commercial value, efforts have been made to engineer a biosynthetic retinol pathway in microbial hosts, such as *Escherichia coli* [19] and *Saccharomyces cerevisiae* [20]. Owing to their chemical properties, retinol and retinoic acid easily decompose when exposed to UV light, high temperature, or in the presence of oxygen [21]. Therefore, retinyl palmitate, which is a more stable esterified form of retinol, has been regarded as an alternative to retinol and used as an active ingredient in pharmaceutical and cosmetic products as a potent anti-ageing agent [22]. So far, retinyl palmitate is available as a product of chemical synthesis; however, the chemical synthesis of retinyl palmitate has drawbacks in terms of low or non-site-specific esterification, the necessity of multistep purification, and extremely corrosive catalysts during esterification [23]. Microbial synthesis of retinyl palmitate is regarded as a promising alternative to chemical synthesis because of the mildness of the chemical reactions involved, efficiency of the catalytic reaction, and inherently high selectivity [24]. Nevertheless, there is no report on the biological synthesis of retinyl palmitate in microorganisms.

In this study, we aimed to reconstruct a retinyl palmitate biosynthesis pathway in a β-carotene-overproducing *E. coli* strain engineered for enhancement of isoprenyl pyrophosphate and β-carotene biosynthesis. High-yield retinyl palmitate was obtained using fed-batch fermentation of retinyl palmitate-producing *E. coli*. Moreover, we evaluated the anti-ageing-related activity of the microbial retinyl palmitate purified from the engineered *E. coli* in human foreskin fibroblasts.

## 2. Materials and Methods

### 2.1. Strains, Media, and Growth Conditions

All the *E. coli* strains used in this study are listed in Table 1. *E. coli* TOP10 (Thermo Fisher Scientific, Waltham, MA, USA) was used for gene cloning and plasmid maintenance, while *E. coli* XL1-Blue (Stratagene, San Diego, CA, USA) served as a host strain for the construction and expression of retinoid pathways. *S. ruber* M31 was purchased from Deutsche Sammlung von Mikroorganismen und Zellkulturen GmbH (DSMZ, Braunschweig, Germany). The Hep3B cell line was a gift from Prof. Wook Kim (Ajou University, Suwon, South Korea). *Enterococcus faecalis* GF2 and *Bacillus subtilis* subsp. *spizizenii* W23 [American Type Culture Collection (ATCC) 6633] were purchased from the Korean Collection for Type Cultures (KCTC, Jeongeup, South Korea). Human foreskin HS68 fibroblasts were purchased from ATCC (Manassas, VA, USA; CRL-1635). *E. coli* cells were grown at 37 °C for cloning and at 30 °C for expressing pathway genes with constant shaking at 250 rpm in Luria-Bertani (LB) and Terrific Broth (TB) medium. Ampicillin (100 mg/L) and chloramphenicol (50 mg/L) were added to the media when required.

### 2.2. Plasmid Construction for the Expression of Retinoid Biosynthesis Pathway Enzymes

Three genes, *blh*_SR_ (locus tag SRU_2210, genBank accession: ABC44773.1), *brp*_SR_ (SRU_0742, genBank accession: ABC43854.1), and *bcox*_SR_ (SRU_0127, genBank accession: ABC44374.1), were amplified using PCR from the genomic DNA (gDNA) of *S. ruber* M31. The PCR products treated with *Xba*I and *EcoR*I were purified and cloned at the corresponding sites downstream of the constitutive lac promoter of pUCM. In a similar way, two genes (LRAT_HS_, genBank accession: AAD13529.1 and CRBP_HS_, genBank accession: AAA60257.1) from complementary DNAs (cDNAs) of Hep3B cells were cloned into pUCM. For the construction of a retinyl palmitate pathway, blh_SR_, CRBP_HS_, and LRAT_HS_ were assembled into low-copy-number plasmid pUCMr with a constitutive lac promoter, a ribosome-binding site (RBS), and a terminator, using the USER^®^ cloning method [28]. The mRNA-stabilizing 26 bp sequences (M1-12, M1-37, and M1-46) [29] were renamed as UTR12, UTR37, and UTR46, respectively, and inserted using PCR between regions of the promoter and RBS of pUCMr, thus yielding the plasmids pUCMr12, pUCMr37, and pUCMr46, respectively. The blh_SR_ gene was subcloned into pUCMr12, pUCMr37, and pUCMr46, resulting in the plasmids pUCMr12-blh, pUCMr37-blh, and pUCMr46-blh, respectively.

### 2.3. Genome Engineering for Increasing Isopentenyl Diphosphate Pools

Four genes—*idi* (*E. faecalis*), *ispA (E. faecalis*), *dxs* (*B. subtilis*), and *dxr* (*B. subtilis*; Figure 1) were amplified using PCR with gDNAs of *E. faecalis* and *B. subtilis*. The PCR products treated with restriction enzymes (Table S1) were purified and cloned at the corresponding sites of pUCM, thereby giving rise to the plasmids pUCM-idi, pUCM-ispA, pUCM-dxs, and pUCM-dxr. Linear DNA fragments, directly integrated into the site of *glvC, yjbI, ilvG*, and *agaVWA* of the *E. coli* genome, were prepared by combining 1) a 50 bp left homology arm sequence (glvC, yjbI, ilvG, or agaVWA); 2) a FRT-Km^R^ cassette; 3) an expression module consisting of a promoter, RBS, gene (*idi, ispA, dxs*, or *dxr*), and terminator; and 4) a 50 bp right homology arm sequence using the USER^®^ Cloning Kit (New England Biolabs, Ipswich, MA, USA) with specific primers (Table S1). Genome integration of the four expression modules of *idi, ispA, dxs*, and *dxr* was performed through one-step homologous recombination using the pRed/ET-mediated recombination method (Gene Bridges, Heidelberg, Germany). Integration mutants were selected on LB agar plates containing 30 μg/mL kanamycin, followed by generation of marker-free strains using an FLP recombinase, which was inducibly expressed in the pCP20 helper plasmid. Sequences of the integration sites in the strains were verified using Sanger sequencing of the isolated gDNAs.

### 2.4. Extraction and Analysis of Retinoids

For analytical purposes, retinoid extraction from *E. coli* cells was performed using an extraction method reported earlier [30], with a slight modification. Briefly, retinoids were repeatedly extracted with 10 mL acetone from cell pellets of *E. coli* until all visible color disappeared from the cell residues. Equal volume of a 5 N NaCl solution was added to the combined acetone extract, following which the pH of the mixed solution was adjusted to 2.0 by addition of 85% phosphoric acid (Sigma-Aldrich, Saint Louis, MO, USA). Next, equal volume of hexane was added into the acidified mixture and mixed well. The upper solvent layer containing retinoids was separated and dehydrated by the addition of 0.1 g of anhydrous sodium sulfate followed by a 20 min incubation. The solution was then completely dried in a Genevac™ EZ2 evaporator (SP Industries, Warminster, PA, USA). The dried residues were resuspended in 1 mL acetone and a 5 μL aliquot was injected into an Agilent 1260 high-performance liquid chromatography (HPLC) system (Agilent Technologies, Santa Clara, CA, USA) equipped with a photodiode array detector (Agilent Technologies, Santa Clara, CA, USA) and a Poroshell 120 EC-C18 column (2.1 × 50 mm, 2.7 μm; Agilent Technologies). The column temperature was maintained at 23 °C, while the flow rate was 0.4 mL/min. Two mobile-phase systems [31,32] were used for gradient elution: mobile phase A (methanol, acetonitrile, and acetic acid, 70.0:30.0:0.1, *v*/*v*) and mobile phase B (acetonitrile, methanol, water, isopropanol, and acetic acid, 60.0:20.0:19.0:5.0:0.1, *v*/*v*). The linear gradient was generated as follows: minutes 0–5, 100% B; minutes 5–6, 100% B to 100% A; minutes 6–28, 100% A; minutes 28–29, 100% A to 100% B; and minutes 29–35, 100% B.

For preparative analysis of retinyl palmitate, retinoids were repeatedly extracted from the cell pellets of *E. coli* (approximately 102 g of wet cells) with 300 mL acetone, separated, dehydrated, and dried as described above. The crude extracts were resuspended in a mixture of petroleum ether and ethyl ether (10.0:0.1, *v*/*v*) and loaded onto a glass column (Φ24/28 mm, 250 mL) packed with 30 g of silica gel 60 (Merck, Darmstadt, Germany). Three 100 mL fractions (colored or colorless) were collected by elution with the same mixture of petroleum ether and ethyl ether in the dark. After confirmation of the presence of retinyl palmitate in each fraction using analytical HPLC, as described above, the concentrated fractions were loaded on a Shimadzu Miniprep Liquid Chromatography (LC) system (Tokyo, Japan) equipped with a photodiode array detector (RID-20A, Shimadzu, Kyoto, Japan) and a YMC-triart C18 semi-preparative column (10 × 250 mm, 5 μm; YMC Co. Ltd., Kyoto, Japan). Two mobile phases (A and B) and a gradient profile were used as described above, except for a flow rate of 3.0 mL/min. The fraction corresponding to peak at 325 nm (retinyl palmitate) was collected, dried, and stored at 20 °C in amber vials filled with N_2_ before further analysis. The mass fragmentation spectra of retinoids were monitored in positive mode on an LC-MS 6150 Quadrupole system (Agilent Technologies, Santa Clara, CA, USA) equipped with an atmospheric-pressure chemical ionization interface. The mass spectrometry (MS) conditions used were as follows: nitrogen nebulizer pressure of 30 p.s.i., vaporizer temperature of 200 °C, corona current of 5.0 μA, nitrogen drying gas at 250 °C and a 7.0 L/min flow rate, capillary voltage of 3.8 kV, and fragmentor voltage of 60 V. The mass fragmentation spectra were monitored in the scan range of m/z 100 to 500 in approximately 1 s. Commercial retinol, retinal, retinyl acetate, and retinyl palmitate were purchased from Sigma-Aldrich and served as references for chemical identification. Proton nuclear magnetic resonance (^1^H NMR) spectra of purified and commercial retinyl palmitate (Sigma-Aldrich, Saint Louis, MO, USA) were recorded in CDCl_3_ using a JNM-ECZ 600 (600 MHz) spectrometer (JEOL, Tokyo, Japan).

### 2.5. Batch and Fed-Batch Fermentation

Batch fermentation was carried out at 30 °C, pH 6.8, and a dissolved oxygen (DO) level of > 30% in a 3.5 L BioFlo 320 bioreactor (Eppendorf, Hamburg, Germany) containing 1.5 L of TB medium (20 g/L glycerol). The DO level was maintained by increasing the agitation rate from 300 to 700 rpm and by supplying air at 1.0 gas volume per unit medium volume per minute (vvm). The pH was maintained at 6.8 by automatic addition of 24% (*v*/*v*) NH_4_OH or 4 N HCl solutions. Fed-batch fermentation was conducted at 30 °C, pH 6.8, and a DO level of > 40% in a 3.5 L BioFlo 320 bioreactor containing 1.4 L of R/2 medium [33], supplemented with 20 g of glycerol as a carbon source. The DO level was maintained by increasing the agitation rate from 300 to 1100 rpm and by supplying air and pure O_2_ gas at 1.5 vvm. The pH was controlled using the method described for batch fermentation above. When the initial glycerol was depleted, a feeding solution containing 500 g/L glycerol, 12 g/L MgSO_4_·7H_2_O, and a trace metal solution (6 mL/L) was periodically added to maintain a glycerol concentration of 0–10 g/L in the media via the DO-stat feeding method. Cell growth was monitored by measurement of optical density at 600 nm (OD_600_) on a SpectraMax^®^ Plus384 spectrophotometer (Bio-rad, Hercules, CA, USA). The concentration of glycerol was determined using an Agilent 1100 HPLC equipped with an Agilent 1100 refractive index detector and an Aminex^®^ HPX-87H column (7.8 × 300 mm, Bio-Rad, Hercules, CA, USA) at a flow rate of 0.7 mL/min with 4 mM H_2_SO_4_ as an isocratic mobile phase.

### 2.6. Cell Viability Assay and Enzyme-Linked Immunosorbent Assay

For the cell viability assay, stabilized HS68 cells were cultured at 37 °C. Dulbecco’s Modified Eagle Medium (DMEM; WelGene Inc., Gyeongsan, South Korea) supplemented with 10% of fetal bovine serum (WelGene Inc.) and 100 μg/mL penicillin and streptomycin (WelGene Inc.) was added to the wells containing 10^4^ cells/well, followed by incubation of the cells in a humidified chamber with 5% CO_2_. After 24 h, the cells were treated with various concentrations (12.5, 25, 50, or 100 μg/mL) of purified or commercial retinyl palmitate and further cultured for 24 h. The EZ-Cytox Reagent (10%) from the EZ-Cytox Cell Viability Assay Kit (Dail Lab Service Co., Cheongwon, South Korea) was added to the treated cells and incubated for 1 h. Cell viability was measured as OD_450_ using an enzyme-linked immunosorbent assay (ELISA) reader (TECAN, Mannedorf, Switzerland). For the procollagen synthesis assay, HS68 cells were seeded at a density of 2 × 10^4^/well in 48-well plates filled with serum-free DMEM supplemented with various concentrations (12.5, 25, 50, or 100 μg/mL) of purified or commercial retinyl palmitate. In parallel, 10 ng/mL TGF-β was incubated with HS68 cells as a positive control. After incubation for 48 h, the culture medium was collected and the amount of secreted type I procollagen was determined using the Procollagen Type I C-Peptide Enzyme Immunoassay Kit (MK101; Takara, Japan).

### 2.7. Reverse-Transcription PCR and SDS-PAGE Analyses

For the reverse-transcription polymerase chain reaction (RT-PCR) analysis of blh_SR_, brp_SR_, bcox_SR_, UTR-12blh, UTR-37blh, and UTR-46blh expression, total RNA was extracted from *E. coli* cells in the mid-exponential growth phase using the Hybrid-R™ RNA Purification Kit (GeneAll Biotechnology, Seoul, South Korea). cDNA synthesis from the total RNA samples was carried out using the ReverTra™ Ace qPCR RT Kit (Toyobo, Osaka, Japan). The RT-PCR conditions used were as follows: 25 cycles of 98 °C for 10 s, 47 °C for 25 s, and 72 °C for 15 s. *cysG* gene encoding siroheme synthase served as a reference gene. Quantitative reverse-transcription PCR (qRT-PCR) was performed to measure the mRNA level of cellular retinoic acid-binding protein 2 (*CRABP2)* in HS68 cells treated with various concentrations (12.5, 25, 50, or 100 μg/mL) of purified or commercial retinyl palmitate. Total RNA was extracted from HS68 cells collected after 48 h of treatment using the RNA Extraction Kit (Bioneer, Daejeon, South Korea). cDNAs were synthesized using the ReverTra™ Ace qPCR RT Kit. qRT-PCR was conducted on a Roche LightCycler^®^ Nano with FastStart™ Essential DNA Probes Master (Roche Diagnostics, Indianapolis, IN, USA), and quantification was carried out using the comparative Ct (ΔΔCt) method. The gene encoding GAPDH was used as a control gene. The conditions used for qRT-PCR were as follows: 45 cycles of 95 °C for 30 s, 60 °C for 40 s, and 72 °C for 10 s. The primers used for RT- and qRT-PCR analyses are listed in Table S1. For sodium dodecyl sulphate-polyacrylamide gel electrophoresis (SDS-PAGE) analysis of blh_SR_, brp_SR_, bcox_SR_, UTR-12blh, UTR-37blh, and UTR-46blh, *E. coli* cells in the mid-exponential growth phase were washed twice with 50 mM Tris-HCl (pH 7.5) and disrupted using sonication. Next, 10 μL of crude protein extracts were separated on a 12% (*v*/*w*) SDS polyacrylamide gel. The gels were subsequently stained with Coomassie Brilliant Blue to visualize the protein bands.

### 2.8. Statistical Analysis

Results are expressed as the mean ± standard deviation of three replicates (*n* = 3). Values of * *p* < 0.05, ** *p* < 0.01, or *** *p* < 0.001 were used to denote significant differences between mean values determined using one-way analysis of variance (ANOVA) and the Bonferroni multiple comparison test done using the assistance of SigmaPlot 12.0 (Systat Software Inc., San Jose, CA, USA).

## 3. Results and Discussion

### 3.1. Construction of the β-Carotene Pathway for Retinol Production

Before reconstructing the retinyl palmitate pathway in *E. coli*, precursor pathways [methyl-erythritol 4-phosphate (MEP) and isopentenyl diphosphate (IPP) pathways] were engineered. Given that the major rate-limiting enzymes of the MEP and IPP pathways are 1-deoxyxylulose 5-phosphate synthase (DXS), 1-deoxyxylulose 5-phosphate reductoisomerase (DXR), isopentenyl diphosphate isomerase (IDI), and farnesyl diphosphate synthase (IspA) [34], the four genes encoding these enzymes were individually engineered to serve as an expression module and then integrated into the genome of an *E. coli* strain (named XIASR). Next, to reconstruct a heterologous β-carotene pathway in XIASR, genes encoding geranylgeranyl diphosphate synthase (CrtE), phytoene synthase (CrtB), phytoene desaturase (CrtI), and lycopene cyclase (CrtY) from *P. agglomerans* were individually engineered to be expressed in plasmid pAC [27], thereby resulting in the plasmid pAC-EBIY. Finally, the β-carotene-overproducing XIASR strain harboring pAC-EBIY was named XB.

### 3.2. Retinol Production Using the Expression of Heterologous β-Carotene 15,15′-Oxygenase

**Figure 2:**
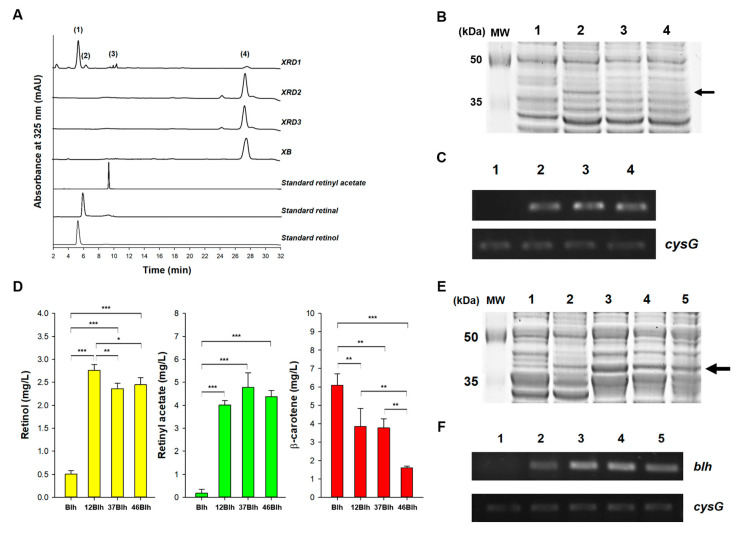
β-carotene 15,15′-oxygenase activity of Blh, Brp, and Bcox from *Salinibacter ruber* in *E. coli* expressing β-carotene and the effect of 5′-UTR engineering in Blh on retinoid production. (**A**) HPLC analysis of acetone extracts of β-carotene-producing XB (a control), blh-expressing XRD1, brp-expressing XRD2, and bcox-expressing XRD3. Peak 1 corresponds to retinol, peak 2 to retinal, peak 3 to retinyl acetate, and peak 4 to β-carotene. Three additional peaks correspond to commercial standards: retinyl acetate, retinal, and retinol. (**B**) SDS-PAGE analysis of expression levels of Blh, Brp, and Bcox. Lane MW represents size makers; lane 1, an empty plasmid (a control); lane 2, Blh; lane 3, Brp; and lane 4, Bcox. The arrow indicates the band corresponding to expressed Blh. (**C**) RT-PCR analysis of mRNA expression of Blh, Brp, and Bcox. Housekeeping gene *cysG* served as a reference. The legend of the lanes is the same as that of the SDS-PAGE experiment (C). (**D**) Quantitative analysis of retinoid production in *E. coli* expressing Blh engineered with UTR12, UTR37, and UTR46. Blh represents a UTR-unengineered gene (a control); 12Blh, UTR12-Blh; 37Blh, UTR37-Blh; and 46Blh, UTR46-Blh. Statistical analysis was performed using one-way ANOVA (* *p* < 0.05, ** *p* < 0.01, *** *p* < 0.001). Data are presented as the mean ± SD of biological triplicates. (**E**) SDS-PAGE analysis of expression levels of UTR12-Blh, UTR37-Blh, and UTR46-Blh. Lane MW denotes molecular weight makers; lane 1, an empty plasmid (a control); lane 2, Blh; lane 3, UTR12-Blh; lane 4, UTR37-Blh; and lane 5, UTR46-Blh. The arrow indicates bands corresponding to expressed Blh. (**F**) RT-PCR analysis of mRNA expression of UTR12-Blh, UTR37-Blh, and UTR46-Blh. Housekeeping gene *cysG* was utilized as a reference. The legend of the lanes is the same as that of the SDS-PAGE experiment (E). UTR, untranslated region; HPLC, high-performance liquid chromatography; SDS-PAGE, sodium dodecyl sulphate–polyacrylamide gel electrophoresis; RT-PCR, reverse-transcription polymerase chain reaction.

Three putative genes (*blh, brp*, and *bcox*) encoding Bcox or β-carotene cleavage enzymes of *S. ruber* were chosen based on an analysis of amino acid similarity to human Bcox. These enzymes have been reported as opsin-like proteins in a previous study; however, they have not been functionally characterized yet [16]. Genes *blh, brp,* and *bcox* were cloned into plasmid pUCMr (resulting in plasmids named as pUCMr-blh_SR_, pUCMr-brp_SR_, and pUCMr-bcox_SR_, respectively) and co-expressed in the β-carotene-overproducing XB strain, thus giving rise to the strains XRD1, XRD2, and XRD3, respectively. After the strains XRD1, XRD2, and XRD3 (as well as the XB strain as a control) were aerobically grown for 48 h in flasks containing TB medium with 2% glycerol, acetone extracts of the four strains were analyzed using LC and LC-MS. Among the four extracts—XB, XRD1, XRD2, and XRD3—one new main peak (peak 1 in Figure 2A) was detected at the same retention time as that of a retinol standard only in case of XRD1, with the same UV/Vis spectrum (Figure S1A,B). LC-MS analysis revealed that peak 1 corresponds to the same molecular fragment with *m*/*z* 269.2 [MH-18]^+^ (lower chromatogram in Figure S1D) as that of the retinol standard (upper chromatogram in Figure S1D). Besides peak 1, two new minor peaks (peaks 2 and 3 in Figure 2A) were detected, with another minor peak corresponding to β-carotene (peak 4 in Figure 2A). Additional analysis of the LC and LC-MS data revealed that minor peak 2 corresponded to retinal (Figure S1A,B), while peak 3 corresponded to retinyl acetate (Figure S1A–C,E). According to the identification of the products, retinal (which is an expected end product of Bcox, Figure 1) was almost completely converted into retinol without a direct activity of heterologous retinal reductase, as proved by the negligible amount of retinal (peak 2 in Figure 2A). A similar unknown endogenous reductive reaction converting retinal to retinol in a heterologous host has been reported [19]. Unexpected formation of retinyl acetate without activity of the corresponding enzyme could also be explained as the case of a reaction converting retinal to retinol. Collectively, only the Blh enzyme showed the functional activity of cleaving β-carotene into retinal in *E. coli* as a heterologous host, and then unknown endogenous enzyme(s) or physiological environments possibly transformed retinal into retinol and further into retinyl acetate. Notably, genes *blh*, *bcox*, and *brp* turned out to be efficiently transcribed; however, only *blh* mRNA was functionally translated in the heterologous *E. coli*, judging by the RT-PCR and SDS-PAGE results (Figure 2B-C). The following reasons may explain why no activities of BRP and BCOX enzymes were observed in *E. coli*, even though adequate mRNA expression was confirmed: 1) improper protein folding caused by abnormal RNA secondary structure [35] or low translational efficiency. Bcox catalyzes the reaction of β-carotene cleavage into 2 molecules of retinal as an end-product. Unexpectedly, the *E. coli* strain expressing Blh produced retinol and retinyl acetate as major end-products with a small amount of retinal. As illustrated in Figure 1, retinol is produced from retinal by a reductive reaction catalyzed by retinal reductase, and retinyl acetate is produced from retinol and acetyl-CoA in an esterification reaction catalyzed by retinol acetyltransferase [19]. According to the cases in which retinal reductase-independent retinol formation from β-carotene has been observed in heterologous hosts [36], we suspected that the non-specific activity of *E. coli* endogenous enzyme(s), such as oxidoreductase (YbbO) [19], accounts for the retinal reductase-independent retinol production in the *E. coli* strain expressing Blh. Similar to the retinal reductase-independent retinol formation, retinyl acetate production may also contribute to the activity of unknown endogenous acetyltransferase(s) in *E. coli*, as reported in another study [37]. Quantitative analysis showed that retinol (0.50 ± 0.1 mg/L), retinyl acetate (0.18 ± 0.2 mg/L), and β-carotene (6.09 ± 0.6 mg/L) were produced in XRD1 (Figure 2D). The retinal concentration was too small to measure under flask culture conditions.

### 3.3. mRNA-Stabilizing Region Engineering of Blh for Enhanced Retinol Production

As the concentration of retinol (a substrate for efficient retinyl palmitate biosynthesis) was low in strain XRD, possibly due to the low amount of cellular Blh, the mRNA-stabilizing region (mRS) approach [38] was adopted for enhancing Blh expression. Three synthetic regulatory sequences (named UTR12, UTR37, and UTR46; Table 1) were chosen and inserted between the promoter and RBS regions in pUCMr, resulting in three synthetic mRS-blh_SR_ expression vectors (pUCMr-12blh, pUCMr-37blh, and pUCMr-46blh, respectively). After the three vectors were transfected into XB, giving rise to strains named XRD4, XRD5, and XRD6, these strains were grown in flasks as described in subSection 3.2. The engineering of all three synthetic mRSs significantly enhanced the concentrations of retinol (4.7–5.5-fold) and retinyl acetate (22.3–26.5-fold) in comparison to unengineered Blh (Figure 2D). The highest retinol concentration (2.76 ± 0.12 mg/L, *p* < 0.001) was achieved with UTR12-blh, followed by 2.45 ± 0.15 mg/L (*p* < 0.001; UTR46-blh), and 2.36 ± 0.11 mg/L (*p* < 0.001; UTR37-blh), suggesting that UTR12 was slightly better than UTR46 (*p* < 0.05) and UTR37(*p* < 0.01). In a reverse order, UTR37-blh produced the highest concentration of retinyl acetate (4.77 ± 0.64 mg/L, *p* < 0.001), followed by UTR46-blh (4.37 ± 0.27 mg/L, *p* < 0.001), and UTR12-blh (4.01 ± 0.19 mg/L, *p* < 0.001). The overall 2-fold increase in the concentration of retinyl acetate compared to that of retinol, indicating that a significant amount of cellular retinol was transformed into retinyl acetate. Next, the effect of the UTR module on the expression of Blh was analyzed by measurement of transcription and translation of Blh. The mRNA levels of UTR12-blh and UTR37-blh were similar and ca. 50% higher than those of UTR46-blh according to RT-PCR analysis (Figure 2E). Notably, the protein expression of UTR12-blh was 2-fold higher than that of UTR37-blh, judging by the SDS-PAGE analysis (Figure 2F), although the mRNA levels of UTR12-blh and UTR37-blh were similar. Altogether, the higher functional expression of Blh through UTR engineering enhanced the cleavage of β-carotene into retinal, which resulted in increased concentration of retinol and retinyl acetate. The three synthetic regulatory sequences, UTR12, UTR37, and UTR46, significantly increased the mRNA and protein expression of Blh, thereby enhancing retinol formation (2.76 ± 0.12 mg/L, *p* < 0.001), which was 5.5-fold higher than that observed with unengineered Blh (0.50 ± 0.1 mg/L). This finding suggests that higher functional expression of Blh can further increase the concentration of retinal and retinol to the reported amounts achieved using *E. coli* [17] and *S. cerevisiae* [18]. Of note, mRS engineering influenced retinyl acetate formation more significantly than retinol formation (26.5-fold for retinyl acetate vs. 5.5-fold for retinol). This phenomenon could be attributed mainly to accelerated conversion of a substantial amount of retinol into retinyl acetate. Therefore, to upregulate and direct retinol into retinyl palmitate biosynthesis, unknown endogenous enzyme(s) catalyzing the esterification of retinol and acetyl-CoA need to be identified and preferably deleted. Notably, although UTR12 showed better performance with respect to the expression of Blh than the other UTRs, the UTR effect might be target gene-dependent. In the original study [29], the relative strengths of the three UTRs based on the β-galactosidase activity were as follows: UTR37 (2.5), UTR46 (1.7), and UTR12 (0.1). Therefore, better expression of Blh by means of UTR12, which has the lowest strength of 0.1, suggests that a balance between genetic expression strength and an active-protein level is important [39] and should be considered as one of the important factors when designing UTRs for target protein expression.

### 3.4. Construction of a Retinyl Palmitate Biosynthesis Pathway

**Figure 3:**
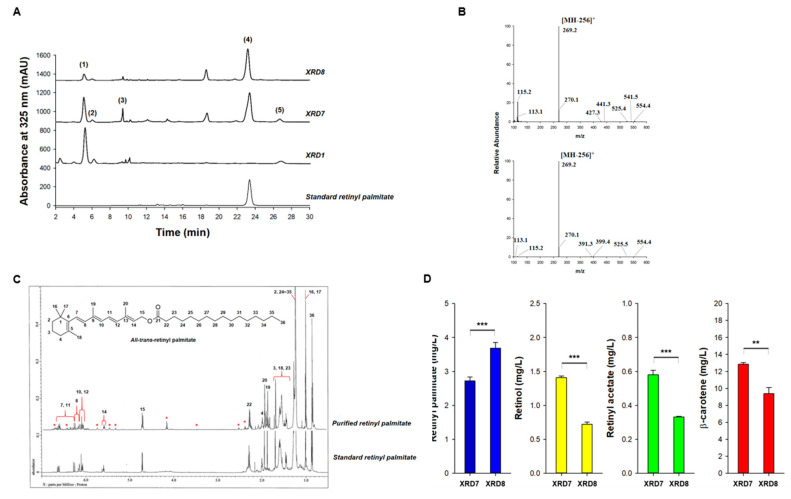
HPLC and LC-MS analyses of acetone extracts of strains XRD7 and XRD8. (**A**) HPLC analysis of acetone extracts of XRD7 (expressing Blh, CRBP, and LRAT) and XRD8 (expressing UTR12-Blh, CRBP, and LRAT). XRD1 (expressing Blh) served as a control strain. Peak 1 corresponds to retinol, peak 2 to retinal, peak 3 to retinyl acetate, peak 4 to retinyl palmitate, and peak 5 to β-carotene. The additional peak corresponds to a commercial retinyl palmitate standard. (**B**) LC-MS analysis of the retinyl palmitate standard (top) and retinyl palmitate (peak 4) purified from XRD8 (bottom). (**C**) ^1^H NMR analysis of the purified retinyl palmitate (upper panel) and the retinyl palmitate standard (lower panel). Asterisks indicate unassigned signals present in the NMR chromatogram of purified retinyl palmitate, in comparison to the retinyl palmitate standard. (**D**) Quantitative analysis of retinoid production in strains XRD7 and XRD8 grown in flasks. Statistical analysis was performed using one-way ANOVA (** *p* < 0.01, *** *p* < 0.001). Data are presented as the mean ± SD of biological triplicates. HPLC, high-performance liquid chromatography; LC-MS, liquid chromatography–mass spectrometry; ^1^H NMR, proton nuclear magnetic resonance.

Next, the retinol biosynthesis pathway was metabolically expanded to retinyl palmitate by co-expression of LRAT [40] and CRBP (Figure 1). Two expression vectors (pUCM-blh_SR_-CRBP_HS_-LRAT_HS_ and pUCM-12blh_SR_-CRBP_HS_-LRAT_HS_) were constructed by assembling CRBP_HS_ and LRAT_HS_ with non-UTR-engineered blh_SR_ or UTR-engineered 12blh_SR_ in the pUCM vector, respectively. Subsequently, each resultant plasmid was transfected into XB, thus, yielding the strains XRD7 and XRD8. LC analysis of acetone extracts of XRD7 and XRD8 cells revealed that a new peak (peak 4 in Figure 3A) was detectable at the same retention time as that of a retinyl palmitate standard in both XRD7 and XRD8. LC-MS analysis (Figure 3B) confirmed that peak 4 corresponded to retinyl palmitate according to the co-occurrence of a molecular ion with m/z 269 [MH-256]^+^, which is a unique fingerprint of the MS fragmentation profile of retinyl palmitate [41], in both acetone extracts and the retinyl palmitate standard. ^1^H NMR analysis showed that the purified retinyl palmitate had an NMR spectrum similar to that of commercial all-trans retinyl palmitate (Figure 3C and Table S2). Eleven unassigned signals (δ 2.11, δ 2.41, δ 2.55, δ 3.49, δ 4.18, δ 5.34, δ 5.48, δ 5.76, δ 5.99, δ 6.36, and δ 6.71) were observed in the ^1^H NMR spectrum of purified retinyl palmitate, which might be from the products of photoinduced degradation of purified retinyl palmitate during isolation and purification (Figure S2). The same ^1^H NMR spectra were obtained on attempting the purification of retinyl palmitate from *E. coli* four times, with utmost precautions to avoid exposure to oxygen and light. As reported in some studies [42], 12 structures of photoinduced-degradation products of retinyl palmitate (Figure S3A–C) were predicted by means of the ^1^H NMR prediction software (Mnova, Mestrelab Research, Spain) and the unassigned 11 signals were identified in the ^1^H NMR spectrum of purified retinyl palmitate (Figure S3D). Quantitative analysis of retinyl palmitate in strains XRD7 and XRD8 revealed that a 1.3-fold better concentration (3.72 ± 0.23 vs. 2.78 ± 0.06 mg/L) (*p* < 0.001) was produced by XRD8 over XRD7 (Figure 3D). These data suggested a positive influence of UTR12 engineering on retinyl palmitate biosynthesis. Nonetheless, considerable amounts of β-carotene accumulated in both the strains (9.56 ± 0.95 mg/L, *p* < 0.01 in case of XRD8 and 12.86 ± 0.26 mg/L in case of XRD7) using flask cultivation, thereby pointing to suboptimal cultivation conditions for retinyl palmitate biosynthesis.

### 3.5. Batch and Fed-Batch Fermentation Procedures for Retinyl Palmitate Production

**Figure 4:**
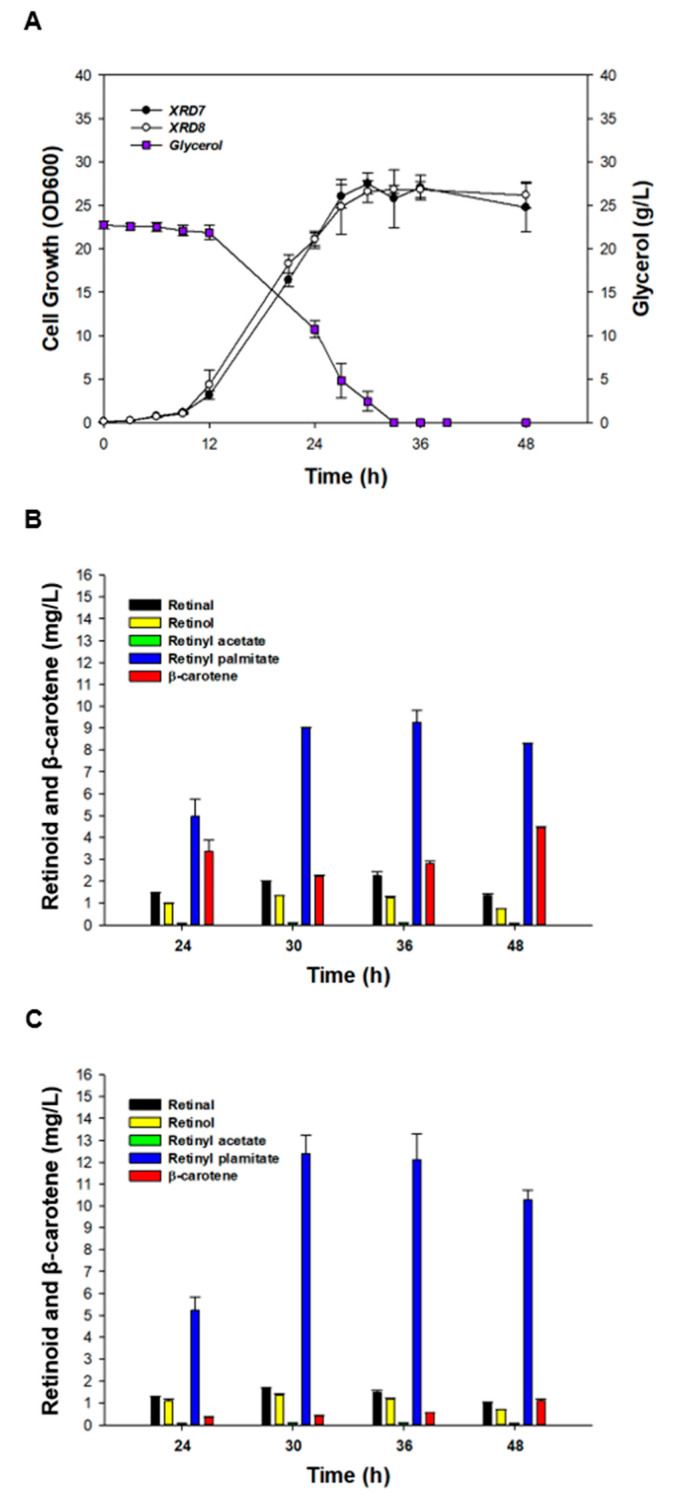
Batch bioreactor fermentation using the strains XRD7 and XRD8. (**A**) Time course of cell growth and glycerol concentration in batch fermentation using XRD7 and XRD8. Concentration of retinal (black), retinol (yellow), retinyl acetate (green), retinyl palmitate (blue), and β-carotene (red) using (**B**) XRD7 and (**C**) XRD8. Error bars are presented as the mean ± SD (*n* = 3).

**Figure 5:**
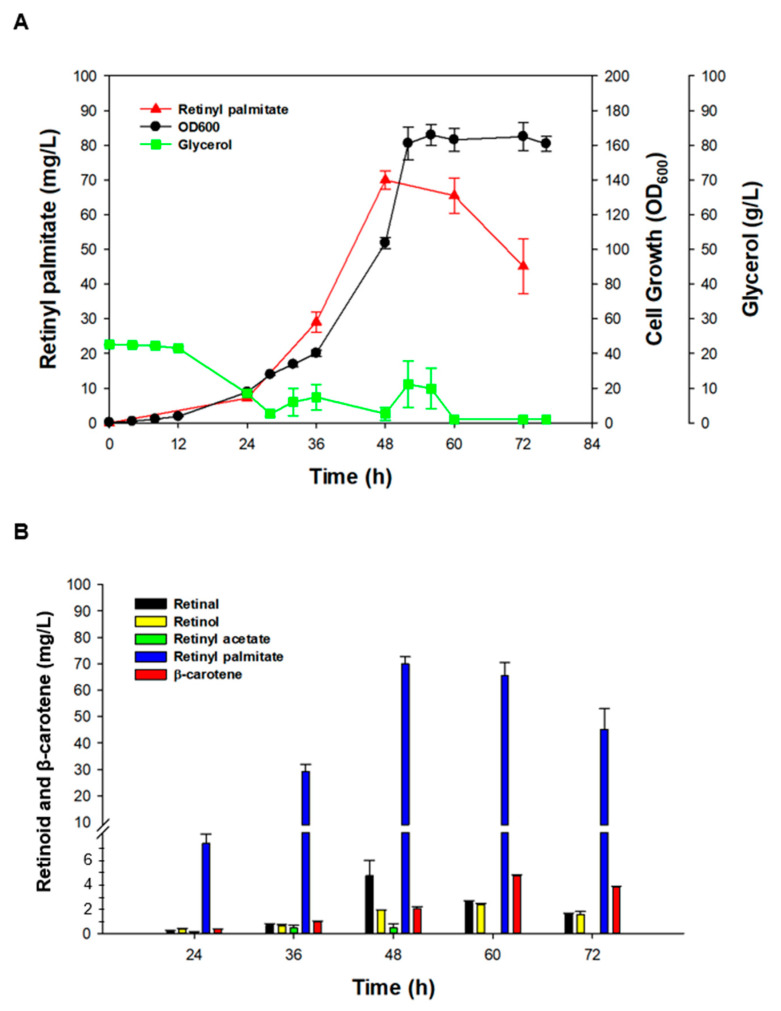
Fed-batch bioreactor fermentation using the strain XRD8. (**A**) Time course of cell growth, glycerol concentration, and retinyl palmitate production during DO-stat fed-batch fermentation in R/2 medium. Black circles represent cell growth (OD_600_); red triangles, retinyl palmitate; and green squares, glycerol concentration. (**B**) Concentration of retinal (black), retinol (yellow), retinyl acetate (green), retinyl palmitate (blue), and β-carotene (red). DO, dissolved oxygen; OD_600_, optical density at 600 nm. Error bars are presented as the mean ± SD (*n* = 3).

To overcome the drawbacks of flask cultures, such as problematic oxygen supply and pH control [43], batch bioreactor fermentation using XRD7 and XRD8 (separately) was carried out with the same composition of media that was used for flask cultivation. Growth and glycerol consumption patterns of the strains XRD7 and XRD8 were similar during bioreactor cultivation (Figure 4A). However, 12.39 ± 0.84 mg/L retinyl palmitate was produced by XRD8, while 9.24 ± 0.58 mg/L retinyl palmitate was generated by XRD7. These levels were 3-fold higher than the yield (3.72 ± 0.23 mg/L, *p* < 0.01) of retinyl palmitate obtained using flask cultivation. The other retinoids were also found to be upregulated in bioreactor cultivation experiments with the strains XRD7 and XRD8: 2.26 ± 0.17 mg/L retinal, 1.24 ± 0.08 mg/L retinol, 0.1 ± 0.01 mg/L retinyl acetate using XRD7; and 1.48 ± 0.10 mg/L retinal, 1.19 ± 0.04 mg/L retinol, 0.1 ± 0.01 mg/L retinyl acetate using XRD8 (Figure 4B-C). Subsequently, to obtain the highest concentration of retinyl palmitate, fed-batch fermentation using XRD8 was performed in R/2 medium. The cells grew to an OD_600_ of 164.4 ± 4.3 in 53 h, after which the cell growth reached a stationary phase (Figure 5A). Retinyl palmitate production increased proportionally to the cell growth until 48 h; the highest concentration of retinyl palmitate (69.96 ± 2.64 mg/L) was achieved after 48 h of culture. Notably, retinyl palmitate production gradually decreased after 48 h and diminished to 45.08 ± 7.91 mg/L (Figure 5A), whereas β-carotene gradually accumulated to 4.56 ± 0.10 mg/L until 60 h, after which the accumulation of β-carotene decreased (Figure 5B). These findings suggest that further optimization of the fermentation procedure and media should increase the current concentration of retinyl palmitate in recombinant *E. coli*. Combined strain improvement, fermentation and medium optimization, and an efficient purification process may facilitate industrial-scale production of retinyl palmitate using microbial cells.

### 3.6. Anti-Ageing-Related Effect of Retinyl Palmitate (Obtained from a Microorganism) on Human Skin

**Figure 6:**
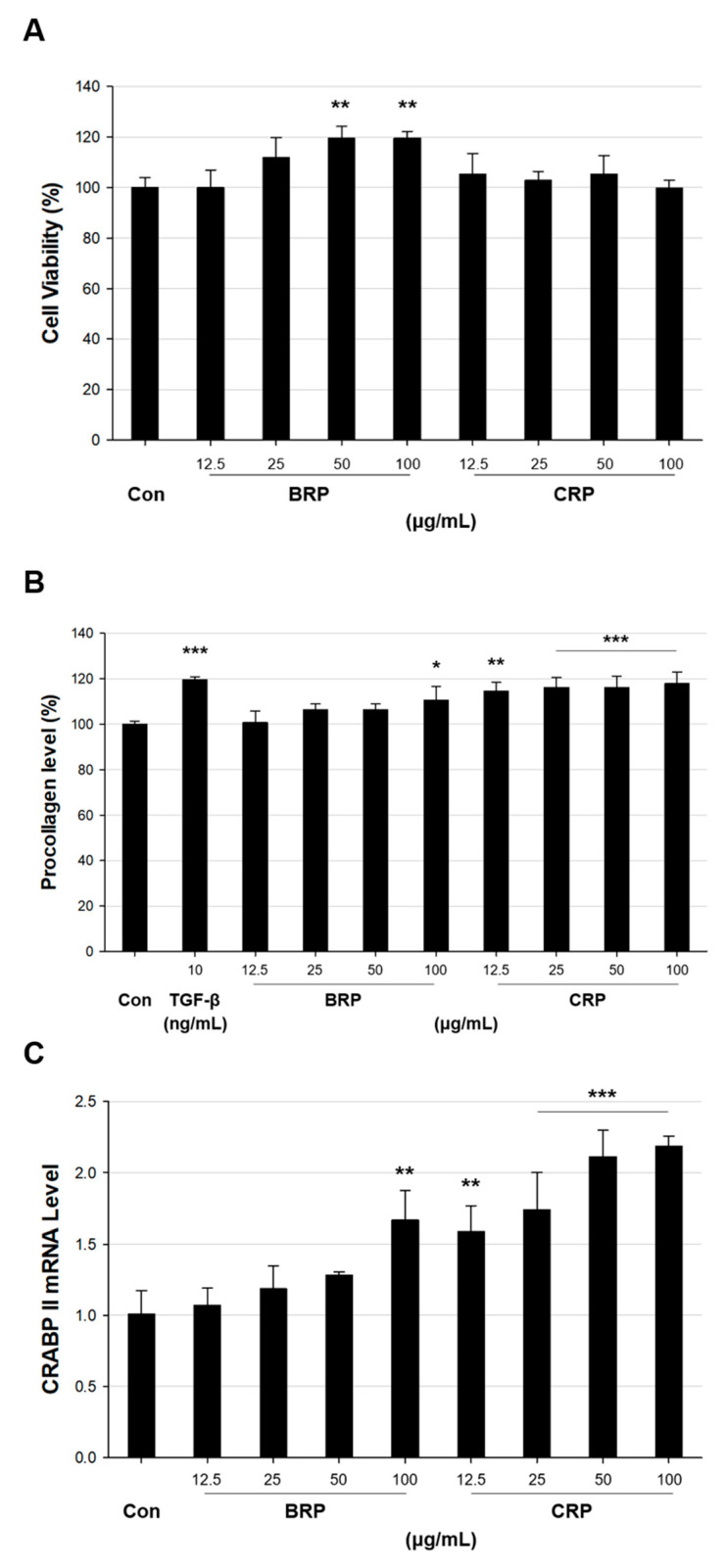
The anti-ageing-related effects of BRP and CRP on HS68 cells. (**A**) HS68 cells were treated with various concentrations of BRP or CRP and further cultured for 24 h. Cell viability was measured as OD_450_ using ELISA. (**B**) ELISA of secreted type I procollagen in cultures of HS68 cells treated with various concentrations of BRP or CRP. TGF-β (10 ng/mL) was incubated with the cells as a positive reference. (**C**) The level of *CRABP2* mRNA in HS68 cells treated with various concentrations of BRP or CRP. Statistical analysis was performed using one-way ANOVA (* *p* < 0.05, ** *p* < 0.01, *** *p* < 0.001 versus a Con group, *n* = 3)). Data are presented as the mean ± SD. BRP, bio-retinyl palmitate; CRP, chemically-synthesized retinyl palmitate; Con, control.

Like other retinoids, such as retinol, retinyl palmitate has a strong anti-ageing activity on human skin and is commercially used as an anti-ageing ingredient in the cosmetics industry. The anti-ageing effect of chemically synthesized retinyl palmitate (CRP) has been proven in human foreskin fibroblasts and even directly in human skin. On the contrary, since there are no microbial/biotechnical alternatives to chemical synthesis, the same effect of so-called “bio-retinyl palmitate” (BRP) has not yet been reported. Therefore, the anti-ageing-related activity of BRP purified from XRD8 was investigated (with CRP as a control) on human foreskin fibroblasts (HS68). When various concentrations of BRP or CRP were incubated with HS68 cells, cytotoxicity was not observed up to a concentration of 100 μg/mL (Figure 6A). Given that the expression of type I procollagen [44] and the mRNA level of *CRABP2* [45] are commonly used measures of cellular effects of retinoids, first, type I procollagen expression in HS68 cells was determined using an ELISA after HS68 cells were treated with various concentrations (12.5, 25, 50, or 100 μg/mL) of BRP or CRP and with 10 ng/mL transforming growth factor-β as a control. Type I procollagen levels increased with increasing concentrations (12.5, 25, 50, and 100 μg/mL) of BRP or CRP (Figure 6B): 111% (*p* < 0.05) at 100 μg/mL BRP vs. 118% (*p* < 0.001) at 100 μg/mL CRP. Next, the mRNA level of *CRABP2* was measured in HS68 cells after treatment with the same concentrations of BRP or CRP as those used in the type I procollagen assay. Similar to type I procollagen, the level of *CRABP2* mRNA increased in a dose-dependent manner in response to increasing concentrations of BRP or CRP (Figure 6C) and reached 1.7-fold (*p* = 0.001) at 100 μg/mL BRP and 2.2-fold (*p* < 0.001) at 100 μg/mL CRP. According to the assay of type I procollagen and *CRABP2* mRNA, BRP clearly showed anti-ageing-related activity similar to that of CRP, although a higher dose of BRP was needed to achieve the same anti-ageing-related effect as that of CRP. This may be explained by the presence of photoinduced-degradation products of BRP (in the sample); these might have lower anti-ageing-related activity than that of BRP. Although we did not investigate other biological activities of BRP, its role(s) in the antioxidant defense system, like the biological activity of retinoic acid in reducing oxidative stress and apoptosis, is worth exploring [46]. As the microbial production of retinyl palmitate is at the early stage of development, the concentration of microbial retinyl palmitate in the current state is not comparable with that of CRP yet. This circumstance can be overcome by further refinements in strain development and optimization of downstream processes. Moreover, rational redesigning of fatty acid biosynthesis pathways in retinyl palmitate-producing *E. coli* could lead to the synthesis of other retinyl fatty acids, including retinyl stearate.

## 4. Conclusions

In this study, a retinyl palmitate biosynthesis pathway was reconstructed in metabolically engineered *E. coli*. We achieved a yield of 69.96 ± 2.64 mg/L retinyl palmitate using fed-batch cultivation. Although the obtained concentration of retinyl palmitate is not sufficient for commercial scale, strain improvements can enhance the comparable concentration of retinyl palmitate. Furthermore, other retinyl fatty acids may be produced by further metabolic engineering of retinyl palmitate-producing *E. coli*. The retinyl palmitate purified from the microbial cells showed similar dose-dependent anti-ageing-related activity toward human fibroblasts as CRP. Taken together, our results clearly show that microbial production of retinyl palmitate is a promising alternative to the chemical synthesis method.

## Figures and Tables

**Table 1:** Bacterial strains and plasmids used in this study.

Strains and Plasmids	Relevant Properties	Source or Reference
Strains		
*E. coli* TOP10	F- *mcrA* Δφ80*lacZ*ΔM15 Δ*lacX*74 *nupG recA1 araD139* Δ(*ara-leu*)7697 *galE15 galK16 rpsL*(StrR) *endA1*	Invitrogen
*E. coli* XL1-Blue	*EndA*1 *gyrA*96(nalR) *thi*-1 *recA*1 *relA*1 *lac glnV*44 F′[::*Tn*10 *proAB*+ *laclq Δ*(*lacZ*)M15] *hsdR*17 (rK- mK+)	Stratagene
*E. faecalis* Gf2	Source for *idi* and *ispA*	KCTC21002
*B. subtilis* subsp*. spizizenii* W23 ATCC 6633	Source for *dxs* and *dxr*	KCTC2189
*S. ruber* strain M31	Source for *blhSR, brpSR,* and *bcoxSR*	DSM13855
Hep3B cell line	Source for *CRBP* and *LRAT*	[25]
HS68	Human foreskin fibroblast cell 68	ATCC CRL-1635
Recombinant *E. coli* strains		
XIASR	XL1-Blue Δ*glvC::idi* Δ*yjbI::ispA* Δ*ilvG::Dxs* Δ*agaVWA::dxr*	This study
XB	XIASR, pAC-*crtEPA*-*crtBPA*-*crtIPA*-*crtYPA*	This study
XRD1	XB, pUCMr-*blhSR*	This study
XRD2	XB, pUCMr-*brpSR*	This study
XRD3	XB, pUCMr-*bcoxSR*	This study
XRD4	XB, pUCMr-12*blhSR*	This study
XRD5	XB, pUCMr*-*37*blhSR*	This study
XRD6	XB, pUCMr-46*blhSR*	This study
XRD7	XB, pUCM-*blhSR*-*CRBPHS*-*LRATHS*	This study
XRD8	XB, pUCM-12*blhSR*-*CRBPHS*-*LRATHS*	This study
Plasmids for pathway construction		
pUCM	Cloning vector modified from pUC19; constitutive *lac* promoter, Amp	[26]
pET21a	Source for *rop*	Novagen
pUCMr	Cloning vector modified from pUCM; constitutive *lac* promoter and *rop* gene, Amp (low copy plasmid)	This study
pUCMr12	Cloning vector modified from pUCMr; UTR12 sequence (GTTTAAACTGACTGACGCACCAAAAG)	This study
pUCMr37	Cloning vector modified from pUCMr; UTR37 sequence (GTTTAAACAATAAATTACGAGCCAGT)	This study
pUCMr46	Cloning vector modified from pUCMr; UTR46 sequence (GTTTAAACCGAATTGGTGGGGCG)	This study
pUCMr-*blhSR*	Constitutive expressed *blh* gene from *S. ruber*	This study
pUCMr-*brpSR*	Constitutive expressed *brp* gene from *S. ruber*	This study
pUCMr-*bcoxSR*	Constitutive expressed *bcox* gene from *S. ruber*	This study
pUCMr-12*blhSR*	Constitutive expressed *blh* gene from *S. ruber* with UTR12 sequence	This study
pUCMr-37*blhSR*	Constitutive expressed *blh* gene from *S. ruber* with UTR37 sequence	This study
pUCMr-46*blhSR*	Constitutive expressed *blh* gene from *S. ruber* with UTR46 sequence	This study
pUCM-*LRATHS*	Constitutive expressed *LRAT* gene from *Homo Sapiens*	This study
pUCM-*CRBPHS*-*LRATHS*	Constitutive expressed CRBP and *LRAT* genes from *Homo Sapiens*	This study
pUCM-*blhSR*-*CRBPHS*-*LRATHS*	Constitutive expressed *blh, CRBP,* and *LRAT* genes	This study
pUCM-12*blhSR*-*CRBPHS*-*LRATHS*	Constitutive expressed 12*blh, CRBP,* and *LRAT* genes	This study
pAC-*EBIY*	Constitutive expressed *crtE, crtB, crtI, and crtY* genes from *Pantoea agglomerans*	[27]
Plasmids for genome engineering		
pUCM-*idi*	Constitutive expressed *idi* gene from *E. faecalis* Gf2	This study
pUCM-*ispA*	Constitutive expressed *ispA* gene from *E. faecalis* Gf2	This study
pUCM-*dxs*	Constitutive expressed *dxs* gene from *B. subtilis* subsp*.spizizenii* W23 ATCC 6633	This study
pUCM-*dxr*	Constitutive expressed *dxr* gene from *B. subtilis* subsp*.spizizenii* W23 ATCC 6633	This study
pFRT-*idi*	Integrative plasmid, *glvC*_UP-FRT-pPGK-*KanR*-FRT-*idi*-*glvC*_down cassette with ColE1 origin	This study
pFRT-*ispA*	Integrative plasmid, *yjbI*_UP-FRT-pPGK-*KanR*-FRT-*ispA*-*yjbI*_down cassette with ColE1 origin	This study
pFRT-*dxs*	Integrative plasmid, *ilvG*_UP-FRT-pPGK-*KanR*-FRT-*Dxs*-*ilvG*_down cassette with ColE1 origin	This study
pFRT-*dxr*	Integrative plasmid, *agaVWA*_UP-FRT-pPGK-*KanR*-FRT-*Dxr*-*agaVWA*_down cassette with ColE1 origin	This study

## Supplementary Materials

The following are available online at https://www.mdpi.com/2076-3921/9/11/1130/s1, Table S1: Primers used in this study; Table S2: Chemical shifts of BRP and CRP in the ^1^H-NMR spectrum; Figure S1: HPLC, UV/Vis, and LC-MS analyses of retinol, retinal, retinyl acetate, and retinyl palmitate in the strains XRD1 and XRD7; Figure S2: Time course ^1^H NMR analysis of the instability of retinyl palmitate under illumination; Figure S3: Predicted structures of photodecomposed retinyl esters, retinyl palmitate isomers, and retinyl palmitate, and prediction of unassigned signals in the ^1^H NMR spectrum of BRP.
