# Supplementary material for: Microbial Production of Retinyl Palmitate and Its Application as a Cosmeceutical
Source: Antioxidants (Basel). 2020 Nov 14;9(11):1130. doi: 10.3390/antiox9111130 (PMC7698207; doi:10.3390/antiox9111130)

Supplementary Material

Microbial Production of Retinyl Palmitate and Its Application as a Cosmeceutical

Bo Hyun Choi [1], Hee Jin Hwang [1], Ji Eun Lee [2], Soon Hwan Oh [1], Jae Sung Hwang [2], Bun Yeoul Lee [1] and Pyung Cheon Lee [1,*]

[1] Department of Molecular Science and Technology, Ajou University, World cup-ro, Yeongtong-gu, Suwon-si, Gyeonggi-do 16499, South Korea; bohyunho0@ajou.ac.kr (B.H.C.), jinilucifer@ajou.ac.kr (H.J.H.), portejuio@ajou.ac.kr (S.H.O.), bunyeoul@ajou.ac.kr (B.Y.L.)

[2] Department of Genetic Engineering & Graduate School of Biotechnology, College of Life Sciences, Kyung Hee University, Yongin-si, Gyeonggi-do 17104, South Korea; jieun0405@khu.ac.kr (J.E.L.), jshwang@khu.ac.kr (J.S.H.)

* Correspondence: pclee@ajou.ac.kr; Tel.: +82-31-219-2461

Table S1. Primers used in this study

Primer Name	Sequences (5′→3′)
[Retinoid biosynthesis pathway construction]	
blh$_{SR}$-F-*Xba* I	GCTCTAGAAGGAGGATTACAAAATGCACAACCCGGTTACCC
blh$_{SR}$-R-*EcoR* I	GGAATTCTCAGGAGACGGCCTGGG
brp$_{SR}$-F-*Xba* I	GCTCTAGAAGGAGGATTACAAAATGGTGGGGTACCTCATCG
brp$_{SR}$-R-*EcoR* I	GGAATTCCTACGGCACGTACCAGATG
bcox$_{SR}$-F-*Xba* I	GCTCTAGAAGGAGGATTACAAAATGGGCGCATGCGATTCC
bcox$_{SR}$-R-*EcoR* I	GGAATTCTCAGTCGAAGAACTGCCCG
LRAT$_{H}$-*Xba1*-F	GCTCTAGAAGGAGGATTACAAAATGAAGAACCCCATGCTGGA
LRAT$_{H}$-R-*EcoR* I	GGAATTCTTAGCCAGCCATCCATAGGAA
CRBP1$_{H}$ -*Xba1*-F	GCTCTAGAAGGAGGATTACAAAATGCCAGTCGACTTCACTG
CRBP1$_{H}$-R-*EcoR* I	GGAATTCTCACTGCACCTTCTTGAATAC
pUCM-F	TCTAGAGCGCCCGGGGA
mRS12-pUCM-R	GTTTAAACTGACTGACGCACCAAAAGCGCTCACAATTCCACACAACA
mRS37-pUCM-R	GTTTAAACAATAAATTACGAGCCAGTCGCTCACAATTCCACACAACA
mRS46-pUCM-R	GTTTAAACCGAATTGGTGGGGCGAGACGCTCACAATTCCACACAACA
pUCN-ori-fr	AGGAAGCGGAAGAGCG
pUCN-ori-r	GAAGATCCTTTGATCTTTTCTA
pET-ori-R	TTGAGATCCTTTTTTTCTGC
pET-rop-F	GGTGCGCATGATCGTG
pUC-USER-1R	ATGCAACUCATTAATGAATCGGCCAAC
pUC-USER-3F	AGACAGUCAATCTGCTCTGATGCC
pUC-sub-USER-1-F	AGTTGCAUCCCGACTGGAAAGCG
pUC-sub-USER-2-F	ATCCATGUCCCGACTGGAAAGCG
pUC-sub-USER-2-R	ACATGGAUATGCGGTGTGAAATACC
pUC-sub-USER-5-F	ATATGCGAUCCCGACTGGAAAGCG
pUC-sub-USER-5-R	ATCGCATAUATGCGGTGTGAAATACCG
pUC-sub-USER-3-R	ACTGTCUATGCGGTGTGAAATACCG
[Genome engineering]	
1. Gene cloning	
idi-gf2-F-*Xab* I	GCTCTAGAAGGAGGATTACAAAATGAATCGAAAAGATGAACATC
idi-gf2-R-*EcoR* I	GGAATTCTTAACGTTTTGCGAAAACAGTG
ispA-gf2-F-*Xba* I	GCTCTAGAAGGAGGATTACAAAATGACGAATTTTAGTCAACAG
ispA-gf2-R-*Nco* I	CATGCCATGGCTATCTCAATTGTAACTGAG
dxs-BS-F-*Xba* I	GCTCTAGAAGGAGGATTACAAAATGGATCTTTTATCAATACAGG
*dxs*_BS-R-*Nco* I	CATGCCATGGTCGTTCTTTCTTTGACGTC
dxr-BS-F-*Xba* I	GCTCTAGAAGGAGGATTACAAATTCATGAAGCAACTCACCATTC
dxr-BS-R-*Xma* I-	TCCCCCCGGGTCCTCAGCTTGCGAGACG
2. Plasmid construction with chromosomal gene manipulation	
USER-FRT-F	AGAAGCGUAATTAACCCTCACTAAAGG
USER-FRT-R	ATGACAGCUCGACTCACTATAGGGC
USER-gene-F	AGCTGTCAUCGCAACGCAATTAATGTG
USER-gene-R	AATGTCGUGTAAGGAGAAAATACCGC
USER-origin-F	ATATGCGAUTTGGTAACTGTCAGACC
USER-origin-R	AGGCATGAUAAAAGGCCAGCAAAAGG
User-*glvC*-UP-F for *idi*	ATCATGCCUCATCTTTTTTTTAAAGATGTGTTC
User-*glvC*-UP-R for *idi*	ACGCTTCUTTTCCACATCCTCTTTTCTC
User-*glvC*-DOWN-F for *idi*	ACGACATUTGTTTTACCGCAAAACTGG
User-*glvC*-DOWN-R for *idi*	ATCGCATAUTGATCCAGCTCTTTTTTTGG
User-*yjbI*-UP-F for *ispA*	ATCATGCCUTTCTTTTCCTTTCGCCG
User-*yjbI*-UP-R for *ispA*	ACGCTTCUCTGTTTAAACCTGGC
User-*yjbI*-DOWN-F for *ispA*	ACGACATUTGAAATCCGTTCTGGC
User-*yjbI*-DOWN-R for *ispA*	ATCGCATAUTCATCTGTTGGGTATCG
USER-*ilvG*-UP-F for *dxs*	ATCATGCCUTGAATGGCGCACAGTGG
USER-*ilvG*-UP-R for *dxs*	ACGCTTCUGCCCGGTTATCAGGTTG
USER-*ilvG*-DOWN-F for *dxs*	ACGACATUCGGTGACGCTATCTACG
USER-*ilvG*-DOWN-R for *dxs*	ATCGCATAUCGTTTTACGGTGCCCAG
USER-*agaVWA*-UP-F for *dxr*	ATCATGCCUAAAGAGGAACACGCTATGC
USER-*agaVWA*-UP-R for *dxr*	ACGCTTCUACCAGCAGGATTTTCTGTC
USER-*agaVWA*-DOWN-F for *dxr*	ACGACATUGATGCTGGTGCCGAC
USER-*agaVWA*-DOWN-R for *dxr*	ATCGCATAUCTTCTTCACCACATAACGTA

[Quantitative analysis of gene transcription levels]	
blh$_{SR}$-RT-F	ATGTCGATGGCCCAGTTC
blh$_{SR}$-RT-R	GGGTCATGATGGCGATCA
brp$_{SR}$-RT-F	TACATGGTGCACATCTTCGG
brp$_{SR}$-RT-R	CGTGAAGTAGACGACCAGG
bcox$_{SR}$-RT-F	ATGCTCTTCCACACGTTGTC
bcox$_{SR}$-RT-R	CACTCCGACAGGATGACG
*CRABP*II-RT-F	GAGGGAGACACTTTCTACATCAAAA
*CRABP*II-RT-R	CCCATTTCACCAGGCTCTTA

Underlined: Shine-Dalgarno sequences.

Table S2. Chemical shifts of BRP and CRP in the ^{1}H-NMR spectrum

Position	Chemical Group	δ H (ppm)		Multiplicity[a]	Coupling Constants *J* (Hz)
		CRP	BRP		
2	*C-CH₂-C*	1.25	1.24	m	-
3	*C-CH₂-C*	1.46	1.46	m	-
4	*C-CH₂-C*	2.01	2.01	t	6.1
7	Olefinic proton	6.29	6.29	d	15.1
8	Olefinic proton	6.16	6.16	d	14.1
10	Olefinic proton	6.12	6.12	d	11.2
11	Olefinic proton	6.63	6.63	dd	15.1; 11.3
12	Olefinic proton	6.09	6.09	d	13.9
14	Olefinic proton	5.61	5.61	t	7.0
15	*CH₂-O*	4.72	4.72	d	7.0
16	*-CH₃*	1.02	1.02	s	-
17	*-CH₃*	1.02	1.02	s	-
18	*-CH₃*	1.71	1.71	s	-
19	*-CH₃*	1.89	1.89	s	-
20	*-CH₃*	1.95	1.95	s	-
22~35	*C-CH₂-C*	1.25	1.24	m	-
36	*-CH₃*	0.88	0.87	s	-

[a]d, doublet; dd, doublet of doublets; t, triplet; s, singlet; m, multiplet.

Figure S1. HPLC, UV/Vis, and LC-MS analyses of retinol, retinal, and retinyl acetate using the strains XRD1 and XRD7. (**A**) HPLC analysis of the acetone extract of XRD1 and XRD7 with authentic standards (retinol, retinal, retinyl acetate, and retinyl palmitate). Peak 1 corresponds to retinol, peak 2 to retinal, peak 3 to retinyl acetate, and peak 4 to retinyl palmitate. Four additional peaks correspond to retinyl palmitate, retinyl acetate, retinal, and retinol standards. (**B**) UV/Vis absorption spectra of retinol (1), retinal (2), retinyl acetate (3), and retinyl palmitate (4) from XRD1 and XRD7 (black) and authentic standards (blue). (**C**) HPLC analysis of the acetone extract of XRD1 with an authentic standard of retinyl acetate. Arrows indicate peaks of retinyl acetate in the acetone extract of strain XRD (top) and a retinyl acetate standard (bottom). (**D**) LC-MS analysis of a retinol standard (upper panel) and retinol (peak 1) that was present in the acetone extract of blh-expressing strain XRD1 (lower panel). (**E**) LC-MS analysis of retinyl acetate in the acetone extract of XRD (upper panel) and a retinyl acetate standard (lower panel). HPLC, high-performance liquid chromatography; UV/Vis, ultraviolet-visible; LC-MS, liquid chromatography–mass spectrometry.

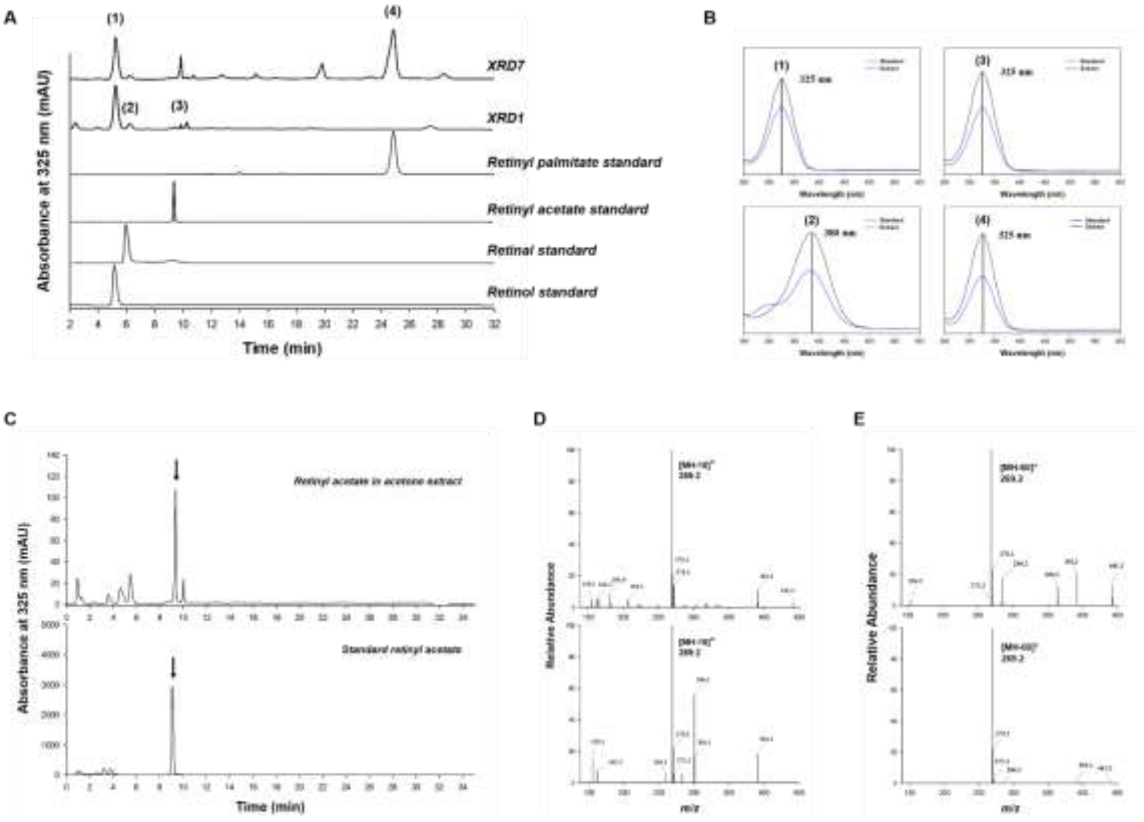

Figure S2. Time course ^{1}H NMR analysis of the instability of retinyl palmitate under illumination. After exposure to light for 72 h, the stability of authentic retinyl palmitate was analyzed using ^{1}H NMR spectroscopy. Red asterisks in purified retinyl palmitate correspond to the progressively developing signals in authentic retinyl palmitate exposed to light. ^{1}H NMR, proton nuclear magnetic resonance

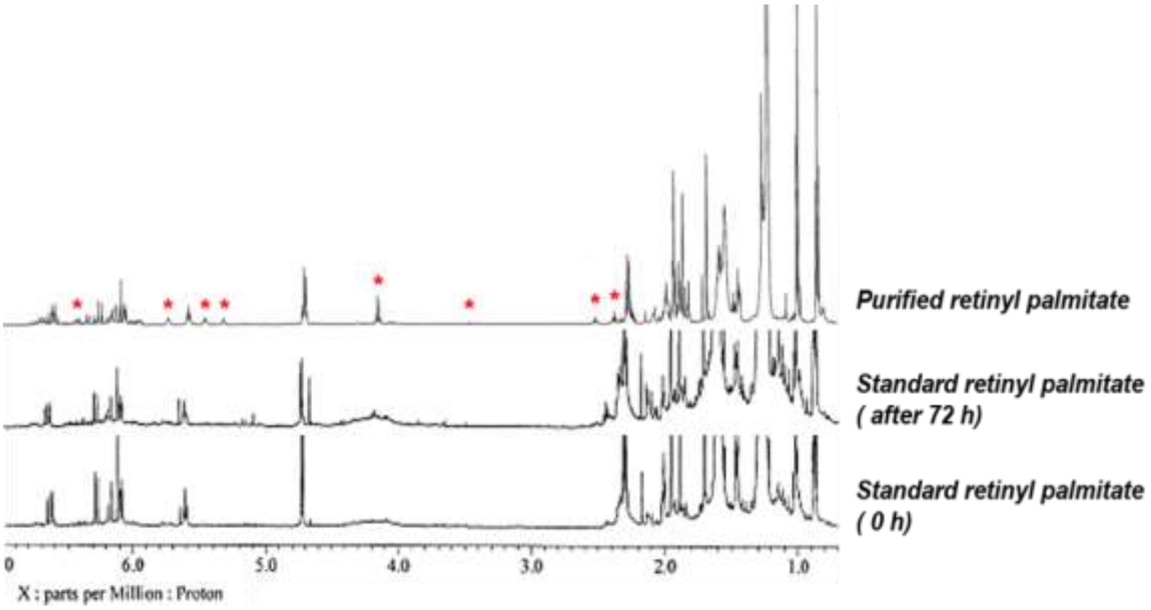

Figure S3. Predicted structures of photodecomposed retinyl esters, retinyl palmitate isomers, and retinyl palmitate, and prediction of unassigned signals in the ^{1}H NMR spectrum of BRP. An ^{1}H NMR prediction tool (Mnova) was used to predict possible ^{1}H NMR spectra of (**A**) retinyl esters, (**B**) retinyl palmitate isomers, and (**C**) photodecomposition products of retinyl palmitate. (**D**) Prediction of unassigned signals in the ^{1}H NMR spectrum of BRP. Blue and red signals represent the ^{1}H NMR spectra of authentic retinyl palmitate (blue) and of retinyl palmitate isomers and photodecomposition products (red), respectively. Red asterisks are unassigned signals in the ^{1}H NMR spectrum of BRP. ^{1}H NMR, proton nuclear magnetic resonance; BRP, bio-retinyl palmitate.

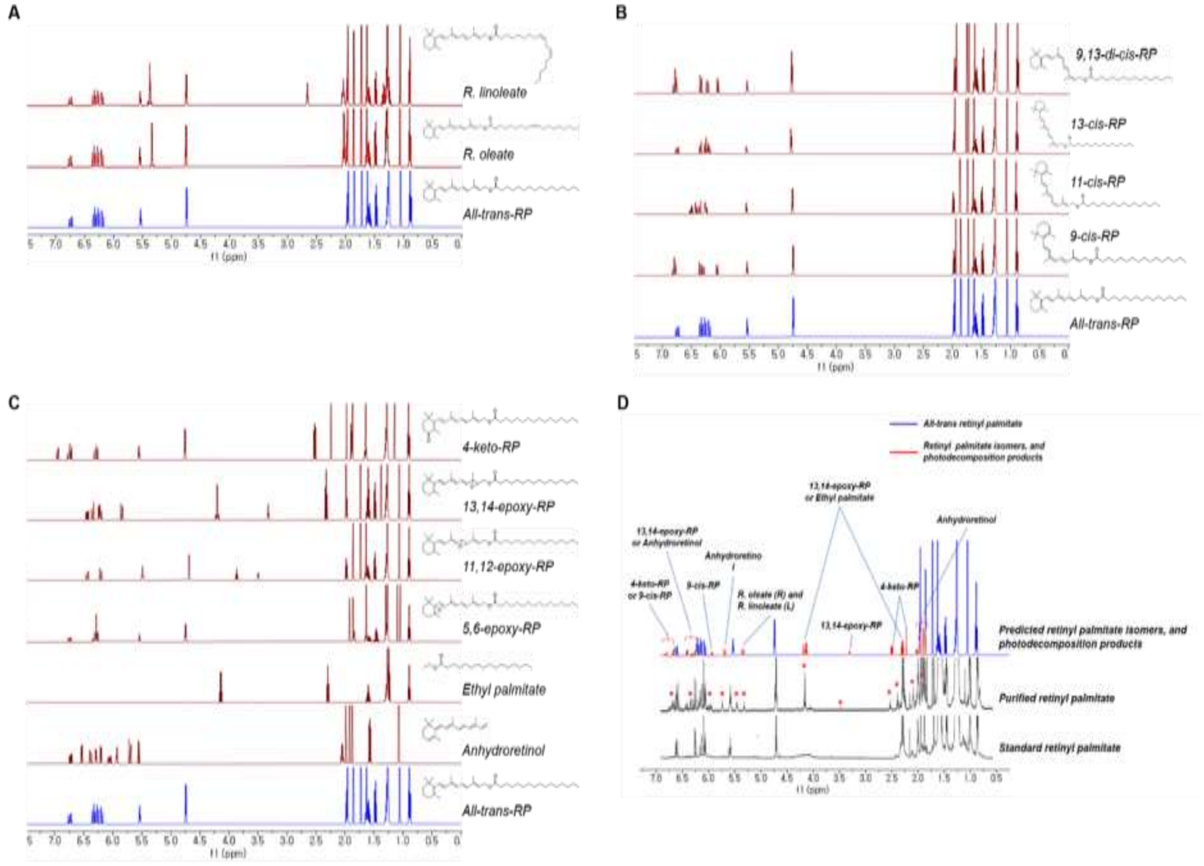

Supplement: Supplementary file 1 [file antioxidants-09-01130-s001.pdf]
